# Data Management Plans in Horizon 2020: what beneficiaries think and what we can learn from their experience

**DOI:** 10.12688/openreseurope.13342.2

**Published:** 2022-02-21

**Authors:** Daniel Spichtinger

**Affiliations:** 1 University Library, University of Vienna, Vienna, 1010, Austria

**Keywords:** data management plans, data management, Horizon 2020, research data management

## Abstract

**Background: **Data Management Plans (DMPs) are at the heart of many research funder requirements for data management and open data, including the EU’s Framework Programme for Research and Innovation, Horizon 2020. This article provides a summary of the findings of the DMP Use Case study, conducted as part of OpenAIRE Advance.

**Methods:** As part of the study we created a vetted collection of over 800 Horizon 2020 DMPs. Primarily, however, we report the results of qualitative interviews and a quantitative survey on the experience of Horizon 2020 projects with DMPs.

**Results & Conclusions: **We find that a significant number of projects had to develop a DMP for the first time in the context of Horizon 2020, which points to the importance of funder requirements in spreading good data management practices. In total, 82% of survey respondents found DMPs useful or partially useful, beyond them being “just” an European Commission (EC) requirement. DMPs are most prominently developed within a project’s Management Work Package. Templates were considered important, with 40% of respondents using the EC/European Research Council template. However, some argue for a more tailor-made approach. The most frequent source for support with DMPs were other project partners, but many beneficiaries did not receive any support at all. A number of survey respondents and interviewees therefore ask for a dedicated contact point at the EC, which could take the form of an EC Data Management Helpdesk, akin to the IP helpdesk. If DMPs are published, they are most often made available on the project website, which, however, is often taken offline after the project ends. There is therefore a need to further raise awareness on the importance of using repositories to ensure preservation and curation of DMPs. The study identifies IP and licensing arrangements for DMPs as promising areas for further research.

## Introduction

### The importance of data and Horizon 2020 data management provisions

Data has been described as the 21st century’s most valuable resource ^1^. Issues related to data are also a priority of the van der Leyen Commission: to leverage the potential of data, the European Commission (EC) published a data strategy in February 2020 ^2^, with the overall aim of creating a single market for the free flow of data within the EU and across sectors ^3^ and, as a first step towards implementation, the EC proposed a Regulation on European data governance (Data Governance Act) in late 2020. Despite these ambitions, however, discrepancies remain amongst EU member states as concerns the maturity of their data economies ^4^. 

In a parallel and interlocked development, *research* data are also increasingly conceptualized as inherently valuable products of scientific research, rather than components of the research process that have no value in themselves ^5^. Consequently, research funders both on the international and on the national level increasingly include requirements for data management (including openness). One of the trend-setters in this regard was the EC, which developed open research data and research data management requirements in its multiannual framework programme for research and innovation, Horizon 2020 (2014 to 2020). In Horizon 2020, the EC initially ran an open research data pilot scheme (ORD Pilot) in selected thematic areas which was subsequently extended to the whole of Horizon 2020 as of the work programme 2017 (under the principle of “as open as possible, as closed as necessary”, i.e. allowing for opt-outs when justified, i.e. allowing for opt-outs when justified) ^6^. A key component is the obligation to create a Data Management Plan (DMP). In recent years, the objective to make data not only open but FAIR (findable, accessible, interoperable and reusable), has been gaining prominence as an important principle for data management and DMPs ^7^. This trend will continue in the new Framework Programme Horizon Europe (2021–2027).

### The DMP Use Case study goals

In the “DMP Use Case” study ^a^ we aimed to identify good practices but also common challenges amongst a number of DMP use cases across different disciplines. Our goal was to support researchers with their DMP obligations throughout their own European projects; furthermore, DMP analysis is an important resource for a variety of other purposes, for instance training activities but also for further scientific research into data management and re-use practices. The study was undertaken from April 2020 to February 2021 on behalf of the EU funded OpenAIRE-Advance consortium by the University of Vienna Library, with support from the OpenAIRE-Advance RDM Task Force. This publication primarily reports the findings of this project; it also feeds into literature about DMP good practice in a broader FAIR ecosystem and on making them machine actionable.

## Methods

There were two main components to the study: a qualitative part, which consisted of an analysis of six DMPs and interviews with six cases studies, and a quantitative part, which involved a manual and automated screening process to establish a white list of DMPs, as well as a survey of the DMP experiences of H2020 projects. These components are described in more detail below.

### Qualitative dimension

This part of the study first contained a qualitative evaluation of six Horizon 2020 DMPs (see *underlying data*). The evaluation was based on a modified version of the example rubric (CC0) from the DART project ( https://osf.io/26b9r/), which was presented during the IDCC 2016 DMP Workshop in Amsterdam. This rubric was augmented by providing a score of 0 to 2 points for each of the 33 categories, resulting in a maximum of 66 points to be scored ^b^. In a second step, an interview guide ( *Extended data* ^8^) was developed and six interviews with key personnel involved in DMPs were conducted ^c^. For each interview a summary document (not a full transcript) was produced, additionally to the initial DMP assessment. The following selection criteria were used to identify the longlist and shortlist of projects to analyse and interview:

• *Balanced thematic representation:* although no European Research Council (ERC) DMPs were analyzed (see below), the ERC classification for its evaluation panels ^9^ was used as a convenient way to establish a thematic grouping. The aim was to have two DMPs represented from each ERC top level area classification, that is two DMPs from Social Sciences and Humanities (SH), two DMPs from Physical Sciences and Engineering (PE), and two DMPs from Life Sciences (LS). Within these areas, the research interests of the staff of the University of Vienna library were taken into account when making the specific selection.

• *Availability of more than one project specific DMP:* we also wanted to assess whether there have been major updates or progress during project duration, which is why we selected Horizon 2020 projects which submitted more than one version of the DMP.

• *Geographic balance:* we wanted to ensure that we analyse DMPs that reflect geographic balance. This was particularly important for the interview selection.

• *Gender balance:* this criterium was primarily of importance when selecting the interview candidates.

Taking into account these criteria, the potential participants were approached via email; the interviews took the form of video calls of about 30 minutes length each. Interviews were conducted with projects from the following specific ERC areas:

• *Social Sciences and Humanities (SH).* Education: systems and institutions, teaching and learning (SH 4_11); Linguistics: formal, cognitive, functional and computational linguistics (SH4_6).

• *Physical Sciences and Engineering (PE).* Web and information systems, database systems, information retrieval and digital libraries, data fusion PE 6_10 (2x).

• *Life Sciences (LS).* LS7_8 (Health services, health care research); LS7_2 Diagnostic tools (e.g. genetic, imaging).

### Ethical considerations

Due to the low risk nature of the qualitative part of the study and the fact that it raised no significant ethical issues, approval from the University of Vienna's ethics committee was not required. Rather, approval of the survey was provided by the scientific supervisor. Each interviewee was contacted before the interview with information about the study and a consent form. Some participants returned the completed consent form. Where this was not done, participants were asked orally for their consent prior to the interview, which was provided in all cases. Consent obtained orally was noted in the internal summary document, which we created for each interview.

### Quantitative dimension

The decision to include a quantitative dimension was based on the large amount of public DMPs that are available from CORDIS. An initial list of public DMPs was obtained from the Commission (CORDA Support) on April 21, 2020. This was then supplemented by the University of Vienna’s IT support by downloading the full list from https://cordis.europa.eu/data/cordis-h2020projects-xlsx.zip on May 18th, 2020. This data was enriched with further information about the projects (programme topics, funding schemes etc from the EU Data portal (data.europa.eu/euodp/de/data/dataset/cordisH2020projects and data.europa.eu/euodp/en/data/dataset/cordisref-data)

Because not all of these could be analyzed in this study, the decision was made to develop a curated collection for future use and re-use. In order to achieve this, a two-step process, combining manual and automated vetting of these DMPs was applied:

*Stage 1 – manual vetting:* with the help of volunteers from OpenAIRE’s RDM taskforce the initial list of 1552 DMPs downloaded from CORDIS was manually screened according to the following questions:

- Is the document in question really a DMP? This screener was added in order to identify and remove documents that had been wrongly classified as a DMP in the system.
- Is the document really public? This screener was added in order to identify and remove documents that had been wrongly classified as public in the system but in fact were set to restricted and/or confidential by the beneficiary.

**Figure 1.**
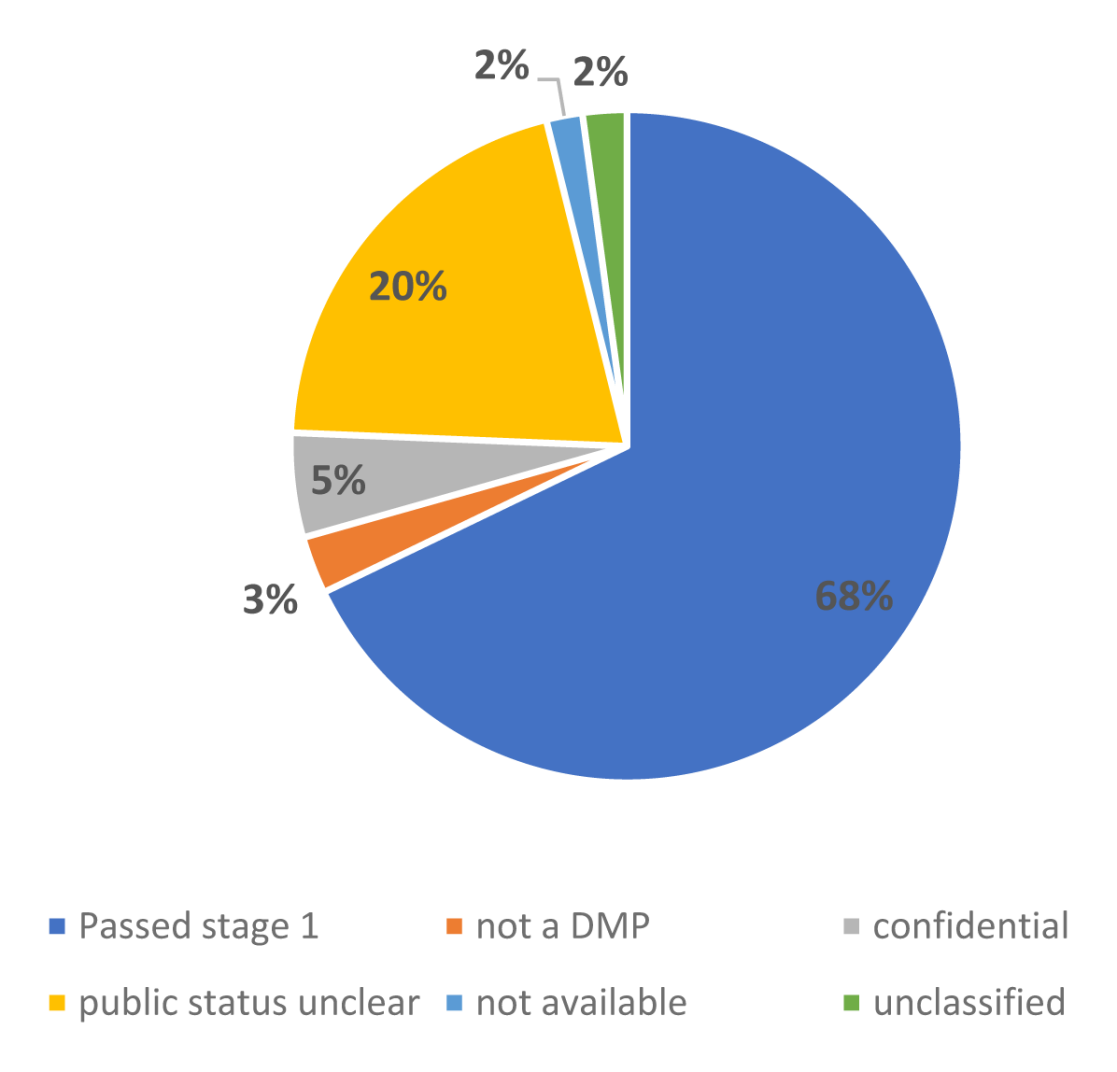
DMP screening stage 1.

In total, 1053 DMPs passed the first stage of the screening: details about these projects , most notably acronym, full title, partners thematic area classification, start and end date, project website URL, project objective, budget, call and funding scheme are available in the supplementary data. For those DMPs that did not pass the first stage, the public nature of the DMP was not clear for 21%, to a significant extent ERC DMPs ^d^. Furthermore, a minority of documents was listed as confidential (5%), followed by 3%, which were not DMPs as well as 2% of DMPs which technically could not be downloaded and another 2% which could not be classified. (see Figure 1).

*Stage 2 – automatic vetting:* an automatic search for the word “copyright” was conducted in each DMP document. Any DMPs that included copyright were excluded from the collection in order to ensure that only those DMPs which are not IP protected are published. It is clear that this processing is not very fine grained and an additional study is currently under way to look at copyright provisions in more detail.

This two-stage process resulted in a list of 840 DMPS which passed both stages. These DMPs are now publicly available for further use (e.g. analysis, training etc) ^10^: https://phaidra.univie.ac.at/detail/o:1140797 ^11^. See *Underlying data* for full details of the final vetted list.

Based on the interview guide for the qualitative component (see *Extended data* ^8^) a survey was developed and distributed to the contact persons indicated on the 840-white listed DMPs via Survey Monkey. This served to further enlarge and broaden the data collection. The survey was filled in by 108 ^e^ projects - 87 of which provided the project acronym and 21 who filled in the survey anonymously. In total, 68 projects were still ongoing at the time of the survey, while 40 were completed (this compares with four completed and two ongoing projects in the qualitative interviews). At the beginning of the online questionnaire, participants were notified that if they proceed this indicates their consent to participate in the research.

## Results

### Knowledge about DMPs prior to the project

In the quantitative survey, 59 projects (54.63%) had been aware of DMPs before their Horizon 2020 project started. A significant minority (49 projects; 45.37%) were thus introduced to DMPs through the Horizon 2020 programme, pointing towards the influence of Horizon 2020 in introducing and spreading the practice of creating a DMP. This is also corroborated by the qualitative interviews: in most of the interviews the participants had been (sometimes vaguely) aware of data management before their Horizon 2020 project, but in several cases the Horizon 2020 project was the first time they actually had to write a DMP. In one case, a project did not in fact participate in the H2020 ORD pilot but still volunteered to do a DMP because they thought it would contribute to a positive evaluation of their proposal. Several interview partners indicated that since their initial involvement their knowledge about data management and DMPs has increased significantly.

### Which work package was/is the DMP part of?

From a project management perspective, anecdotal evidence suggested that the DMP has been integrated into different parts (Work Packages or WPs) of Horizon 2020 projects, notably the general project management WP or the dissemination WP. In most of the qualitative interviews, the DMP been dealt with in the management WP. Given that 51.4% of surveyed projects consider this the appropriate setting for the DMP (see Table 1), there is an indication that this is becoming more standard practice, as DMPs are becoming more widespread. This said, in one project, data management was formally part of the management WP, but informally spread over three WPs related to data acquisition and analysis. In another case DM was split between the management and the dissemination WP. In the quantitative survey, the second most popular work package for the DMP was dissemination (21.5%), followed by a distinct WP solely for data management (17.76%). The latter figure is surprisingly high and not corroborated by the qualitative interviews, in which not a single project had a dedicated DM WP.

**Table 1.** Which work package was/is the DMP part of?

Answer choices	Responses	
part of the project management work package	51.40%	55
part of the dissemination work package	21.50%	23
own work package for data management	17.76%	19
other	9.35%	10
	Answered	107
	Skipped	1

### Feedback from partners

**Figure 2.**
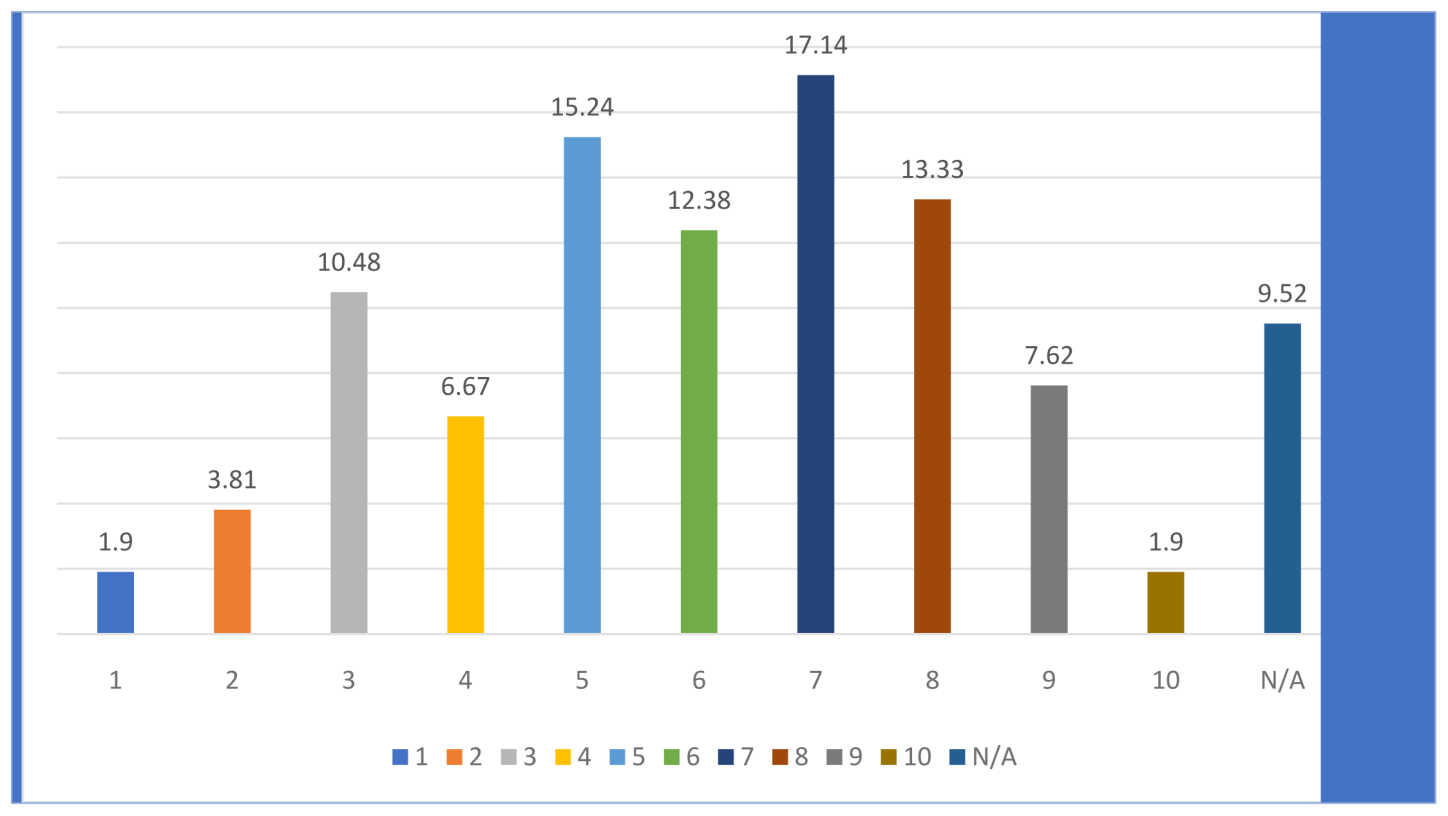
How easy/difficult was it to obtain feedback from the partners for the DMP? (1-very easy, 10 very difficult), numbers in %. *N/A = did not consult with partners N= 105*.

There were a lot of different answers concerning the ease or difficulty of obtaining feedback from project partners in the process of creating and/or updating the DMP, which is perhaps not surprising given the different size and composition of H2020 projects. In the quantitative survey, only a tiny minority thought this was very easy or very difficult, with most responses on the scale of a difficulty of 5–8 (where 1 is very easy and 10 very difficult) see Figure 2. In the qualitative interviews, challenges encountered with other partners concerned:

(i) personal data and GDPR,

(ii) the amount of time and resources needed and

(iii) coordination among geographically distant partners (though this is not necessarily limited to DM)

 One interviewee also stated that ease and quality of feedback depended on the type of data. In the same project there was at least one person per partner involved in data management. In one project it was decided to sign user agreements, with the data belonging to those users but the project having limited usage rights. One participant also stressed that data management does not necessarily mean open: only some data in their project was opened for scientific conferences (e.g. deposited on Zenodo).

### Use of templates & online tools

In the quantitative survey, 40% of the respondents (42 projects) indicated that they used the templates from the EC or the ERC, while 17.14% used another template and 8.57% used a digital tool (see Table 2). From the comments it became apparent that the most often used external tool is DMP online ^f^ from the Digital Curation Center. In total, 25% of respondents did not use a specific tool or template at all but developed their own template. In fact, in the qualitative interviews several interviewees stated that at the time of their project start the EC template was not yet available. Therefore, these projects did their own - sometimes extensive - background research or based their DMP on previous knowledge. Some also partially used the EC template and augmented it with information from other sources and their communities. In one case the library was involved in assisting with the DMP. In another case it was reported that each partner contributed to their own part of the DMP, while in a more recent project of the same partner this was changed to a more unified approach. 

**Table 2.** Did you use a template or online tool when creating the data management plan for your project?

Answer Choices	Responses	
YES- template from the European Commission/European Research Council	40.00%	42
YES - template from another organisation	17.14%	18
YES - online tool	8.57%	9
NO	25.71%	27
not applicable	8.57%	9
If you used a template or online tool from another organisation please specify which template or tool?		26
	Answered	105
	Skipped	3

### Support

When asked whether they received support when creating their DMP, the majority of those surveyed indicated that they received support from other partners (39%), and 27% indicated that they did not receive any support. For the rest, several projects (11.5%) indicated that they received support from the library, 3.8% from OpenAIRE and 2.9% from the IT department (see Table 3). A more fine-grained picture emerges from the qualitative interviews: here one partner mentioned support from the university’s data protection officer and technical input as regards data security. OpenAIRE was also explicitly mentioned several times, as was the advantage of having partners with experience in this area. The library and a data archive was also mentioned in one interview. It was stressed that it would be helpful to have a designated contact at the EC for inquiries.

**Table 3.** Did you receive support when creating your data management plan?

Answer Choices	Responses	
YES- from other partners	39.42%	41
YES-from the library	11.54%	12
YES-from the IT departement	2.88%	3
YES- from OpenAIRE	3.85%	4
NO	26.92%	28
YES Other - please specify	15.38%	16
	Answered	104
	Skipped	4

### Feedback from the EC/the Agency

A total of 55.8% of survey respondents did not receive feedback from the EC or the implementing agency, while 22.1% received feedback from the Project Officer, and 22.1% received feedback from the reviewers (see Table 4). The comments indicate that those that did receive feedback largely considered it useful for their further work. Within the qualitative interviews, none of the six interviewees received any content related feedback from the Commission or Agency but several received feedback from their reviewers. In one case it was mentioned that the Commission itself seemed unsure on how to handle this deliverable.

**Table 4.** Was there feedback from the European Commission/the Agency you submitted the plan to?

Answer Choices	Responses	
YES from the Project Officer	22.12%	23
YES from the Reviewer(s)	22.12%	23
NO	55.77%	58
	Answered	104
	Skipped	4

### DMP project specificities (qualitative interviews only)

Within the qualitative interviews we were able to delve deeper into some of the project specific DMP related issues the interviewees encountered:

• *GDPR compliance:* with older projects this was initially not an issue but became relevant once the GDPR entered into force. One early DMP dealing with privacy issues included a privacy impact assessment; the data in this DMP was not in fact open: “the objective was to be accountable, not open”.

•     One project was concerned with vulnerable groups and therefore has a strong focus on personal data consent forms, data security and ethical issues in their DMP. 

•     One DMP did not consider Creative Commons very useful – the data was not considered an original work in the sense of the German word “Urheberrecht”.

•     One project primarily used pre-existing open data. There was therefore no problem in using an open license – however, the business partners in the project were somewhat critical and saw open data more as an obstacle, rather than as an opportunity.

•     One project illustrates the progressive evolution of DMPs from one version to the next, with some questions only being able to be answered in the final iteration of the document (while in other project DMPs there is little change over time)

•     One project explicitly mentions the lack of community standards as a major barrier.

### Major challenges (qualitative interviews only)

The following major challenges were raised by the interviewees in the qualitative interviews:

•     reading and analyzing partner input and turning it into one understandable document, in particular at the beginning of the project, when there was little experience

•     where to put the focus and how much details to give – internal procedures or output; also whether to tackle any data or data underlying publications (the latter strongly preferred)

•     understanding the technicalities

•     how to create the DMP from scratch with zero experience

•     understanding the requirements and convincing partners to submit thorough information (done through peer pressure). This is easier in newer projects since DMPs are more accepted

•     covering all partners, some of them in non-EU countries where different national policies apply (e.g. on protecting vulnerable groups)

### Usefulness of the DMP, beyond it being an EC requirement

Anecdotal evidence suggested that DMPs may be considered a tick boxing exercise or an unnecessary burden by at least some of the Horizon 2020 beneficiaries. It was therefore somewhat surprising that the survey respondents as well as the interviewees did not share this view ^g^. A total of 53.3% considered the DMP useful beyond it being a EC requirement and an additional 29% considered it somewhat useful, resulting in 82.2% with a generally positive attitude. Only 17.8% did not consider a DMP useful (see Table 5). There were, however, a number of diverging views in the comments. This rather positive view, with some caveats, was also present in the interviews, where the interview partners had the following to say on the usefulness of the DMP beyond it being an EC requirement:

**Table 5.** Do you consider the development of a data management plan useful beyond it being a requirement from the side of the European Commission?

Answer Choices	Responses	
Yes	53.27%	57
Partially	28.97%	31
No	17.76%	19
if you chose "partially", can you specify?		31
	Answered	107
	Skipped	1

•     We turned something that was initially a chore into a Socrative work and learned a lot from it

•     Not every project needs a DMP; a simple checklist would suffice

•     It's challenging having to write a DMP through a pre-existing template because you need to fit your project to pre-existing guidelines. At the same time, it is also useful because during the work one can lose sight of FAIR data and the exercise reminds you to remain on track. A more advanced project might benefit from having its own template though-

•     Very important to be done for each project (regardless of EU funding) but needs different approaches and categories based on the size and the nature of the project (currently not much of a distinction whether it is a project with 500 partners with a lot of shared data or 5 “friends and family”)

•     The only thing it was useful for was to clarify in project meetings which datasets we were talking about. For the overall objective of the project a DMP was not very important

•     DMPs are very useful, also for projects which deal with vulnerable and marginalized groups and long-term curation and preservation. We are switching from destroying data to archiving data after the project end. 

### Publication of the DMP

Interestingly, most of the qualitative interviewees were initially not sure whether their DMP had been published somewhere (except it being submitted to the EC). After checking, many stated that it had been published on the project website, which, however, was no longer online in some cases. In one instance the project was contacted by OpenAIRE and uploaded the DMP to OpenAIRE (alongside the other project deliverables). In the survey, the project website was also the most popular location for publishing the DMP (30.5%); however, the majority of projects did not publish their DMP at all (38.1%). Only 22.9% deposited their DMP in a repository ( Table 6).

**Table 6.** Did you publish your data management plan somewhere?

Answer Choices	Responses	
YES - in a repository	22.86%	**24**
YES - on the project website	30.48%	**32**
YES - somewhere else	8.57%	**9**
NO	38.10%	**40**
If you chose the answer "yes - somewhere else", please specify where		**13**
	Answered	**105**
	Skipped	**3**

### Time and resources

Both the survey respondents and the interviewees struggled with this question and a number of different answers were given. One user pointed to the fact that the existence of a template has made it easier and less resource intensive to create a DMP. Other good advice shared related to spending time on data management in the planning phase, which makes it easier to implement once the project has started (“something well planned is half done”). The normalization and routinization of DMPs also makes it less resource intensive.

As to the time and effort needed, no uniform answer emerged, which is perhaps not surprising given the different size of Horizon 2020 projects and the different thematic areas covered. The same holds true not only for data management plans but also for data management more generally.

### What kind of support is needed and who should provide it?

In contrast to an earlier question that was designed to elicit where support *currently* comes from, this question was designed to elicit where it *should* come from and who should deliver such support. In the qualitative interview the following issues were raised:

•     Reference contact in the Commission to provide training and advice

•     A support paper which contains the requirements from the EU as concretely as possible – a matrix then just needs to be applied. Research support organizations should execute that, single researcher should have an overview and operational support

•     Sustainability questions are important, including how to pay for data management after the end of the project; what are the limits to make data FAIR but at the same time sustainable. Larger infrastructures (ERICs) can help

•     The best approach is to have someone in the data community with expertise to help; the data community should be more approachable for everyone

•     For bigger organizations the library can provide support (and sometimes also the data archive) 

### Final thoughts from the interviewees

The interviewees were provided with the opportunity to flag up any other issues they would like to mention in the context of DMPs and data management. The following aspects were mentioned in the qualitative interviews:

•     The need for awareness raising (in particular as concerns legal regulations for personal data)

•     Taking issues connected with AI into account (very new and not always included in DMPs)

•     Zenodo is a useful tool and collaboration with OpenAIRE works well – we need sufficient political will to continue that

•     When project ends data tends to disappear, people save data in different repositories which make it very dispersed – the best solution would be to have one repository, although a monopoly can also pose problems. We may need a global agreement to releasing open data (COVID could be an opportunity)

•     We often fail at longevity both as concerns tools and repositories (will they still be here in 5 years?) – they don’t always allow you to take data out in accessible formats in an easy way

•     Templates are very useful; it helps to think about data collection but also use (even after project end) 

## Discussion and recommendations

For a significant number of projects, Horizon 2020 was the first time they had to develop a DMP. This underlines the importance of the Commission’s policy and its impact in using the framework programme to promote research data management practices. In a wider context, it generally illustrates the importance of funders to provide clear and ambitious open science requirements in their programmes. Results from the qualitative interviews also indicated that the Horizon 2020 DMP development process has been a learning journey for many of the interviewees: several indicated that they have significantly developed their knowledge on data management, underlining the fast development of data management practices in recent years. Importantly, 82% of survey respondents see data management as useful or partially useful beyond it being just an EC requirement.

As regards project management, having a DMP as part of the WP on management (as opposed to e.g. dissemination) seems to be the most widespread practice, in particular for those projects which do not have data science as their focus. We would therefore generally recommend projects to follow this approach, if there are no good reasons to do otherwise, but at the same time to also ensure links with dissemination activities. In general, having one person per project partner responsible for data issues is a good practice (except for small projects or coordination and support actions where no data is generated). However, there also needs to be a person that takes overall responsibility for the project DMP, so that it is not simply a collection of input delivered by partners but forms a coherent whole.

Templates are clearly important: 40% of the survey participants used the EC and ERC template and these templates seems to have helped to dispel some confusion at the beginning of the H2020 ORD pilot when there was little information available and projects had to do an enormous amount of research themselves. However, some ask for a more tailor-made approach, since one DMP template may not fit all the different kinds of projects funded under Horizon. This could be done through an EC online system for creating DMPs (and not just a pdf template) or through the further development of existing tools such as ARGOS ^h^, which could then be endorsed by the Commission. For details about potential improvements of the template the reader is also referred to the specific OpenAIRE/FAIR Data Expert Group report on the Horizon 2020 template ^12^.

Support in creating DMPs was in most cases received through the project partners (if at all); in some cases the library or a data archive or OpenAIRE were also mentioned as sources of support. In the qualitative interviews, none of the participants received content feedback on the DMP from the Commission or Agency but some did receive feedback from the reviewers. Similarly, in the qualitative survey, the majority of respondents (55%) did not obtain feedback from either, but those that did, found it helpful. Especially beginners report a feeling of being lost and, in particular before the template was available, had to do a significant amount of self-learning (qualitative interviews). A number of interviewees ask for a dedicated contact at the EC to help with the data management plan. I would therefore recommend to set up a “EU one-stop-shop for Horizon research data management”, akin to the IP helpdesk (e.g. through a public procurement procedure or a grant to a named beneficiary). OpenAIRE would be an institution with a lot of knowhow to run such a one stop shop, potentially as part of the European Open Science Cloud (EOSC), which OpenAIRE is an important part of. This should also include further guidance on resources and costing ^13^. This one stop-shop could also train existing local staff as multipliers to support researchers.

Both respondents in the qualitative interviews and the quantitative survey point towards the project website as the main place where they published their DMP. This is somewhat problematic, since project websites tend to be shut down after project end and thus the DMPs (and other deliverables) are not available for long term preservation and curation. On the one hand this points to the importance of CORDIS/CORDA as a source for public DMPs (and other public deliverables). However, there is also a need to further raise awareness of the need to deposit DMPs in repositories to ensure preservation. The deposition process can be combined with assigning a persistent identifier to a DMP, which improves their findability. Moreover, there is also a need to ensure that DMPs themselves are clearly licenced, preferably with a creative commons licence. As Horizon DMPs are classified as deliverables, it is currently up to the consortium whether DMPs are provided open access or not. Projects should be encouraged to apply the “as open as possible, as closed as necessary” principle to DMPs as well.

This study has also identified a number of areas for further research. As concerns the establishment of the DMP white list, a number of DMPs were not included in the list due to their mentioning “copyright” (see Methods section). DMPs would merit closer analysis on what exactly licensing arrangements and any copyright restrictions are - in some cases first evidence indicates that this can be confused, Furthermore, an analysis of ERC DMPs - not included in the curated collection due to the fact that they do not mention whether they are public or not - would also be interesting.

The results reported in this publication and the data underlying it could be useful for the ongoing work of the research data management community, such as the “Exposing Data Management Plans WG" and/or the Active Data Management Plans IG" of the Research Data Alliance (RDA). ^i^ Furthermore, this study also contributes evidence important for funders and policy makers: as more and more funder mandates for data management move from aspirational to hard requirements ^14^, it will be important to monitor in detail not only the uptake but also the experience of the project beneficiaries with current and new requirements. These funder requirements have real live impact on what researchers are required to do with underlying research data and this in turn also has implications for those institutions and organisations that take care of research data. However, rather than seeing funder requirements as an administrative burden they should be considered a strong incentive for change towards a better managed and more transparent scientific system.

## Data availability

### Underlying data

PHAIDRA: Horizon 2020 DMPs what beneficiaries think and what we can learn from their experience (“DMP Use Case Project”), https://phaidra.univie.ac.at/detail/o:1165751 ^8^. Data are available under the terms of the Creative Commons Attribution 4.0 International license (CC-BY 4.0)

This project contains the following underlying data:

a) Individual DMP analysis

- DMP analysis EURHISFIRM (Data management plan analysis of the CORDIS project: EURHISFIRM)
- DMP analysis Edu MAP (Data management plan analysis of the CORDIS project: Edu MAP)
- DMP analysis FREME (Data management Plan analysis of the CORDIS project: FREME)
- DMP analysis READ (Data management Plan analysis of the CORDIS project: READ)
- DMP analysis AFRI ALLIANCE (Data management Plan analysis of the CORDIS project: AFRI ALLIANCE)
- DMP analysis Apollo (Data management Plan analysis of the CORDIS project: Apollo)
- DMP analysis CAREGIVERSPRO-MMD (Data management Plan analysis of the CORDIS project: CAREGIVERSPRO-MMD)
- Evaluation rubric for DMP analysis
- Responses summary (Results of quantitative survey of “DMP Use Case Project”)

b) Interview Guide

c) Collection of vetted DMP

- DMP Case Study Stage 1 Manual Vetting Project DMP List
- DMP Case Study Stage 2 Copyright Vetting Final DMP List
- Individual DMPs at PHAIDRA: DMP Use Case Project, https://phaidra.univie.ac.at/o:1140797 ^11^ -The DMPs available in the collection are clearly marked as public documents (designation PU) and not copyrighted (as far as the latter information could be verified through automation). The CORDIS license allows re-use (see https//cordis.europa.eu/about/legal). Nevertheless, users of the collections are advised to consult the individual DMPs for any restrictions in re-use; PHAIDRA and OpenAIRE Austria accept no liability. The *content* of the DMPs has not been quality reviewed – they are published as is and should not necessarily be taken as good practice cases.
