# Supplementary material for: Data Management Plans in Horizon 2020: what beneficiaries think and what we can learn from their experience
Source: Open Res Eur. 2022 Feb 21;1:42. Originally published 2021 Apr 23. [Version 2] doi: 10.12688/openreseurope.13342.2 (PMC10446041; doi:10.12688/openreseurope.13342.2)
Supplement: Peer review history

# Peer review: Reviewer response for version 1

**DOI:** 10.21956/openreseurope.14412.r27181

**Published:** 2021-08-25

**Authors:** Daniel Mietchen

**Affiliations:** 1 School of Data Science, University of Virginia, Charlottesville, VA, USA

**General comments**

 This study provides a useful introduction to practical aspects of Data Management Plans (DMPs) in the context of Horizon 2020-funded projects and presents an analysis of associated trends with respect to data management more broadly, particularly in the context of Horizon Europe. While the overall conceptualization is solid, the description of the methodology would benefit from greater detail (e.g. sharing of the code involved), as would the discussion of policy implications for key stakeholders like projects, research fields, institutions or the European Commission.

 I read the reviews by Ron Dekker ^[1]^ and by Daniela Adele Hausen & Giacomo Lanza ^[2]^, who expressed much of what my comments would be. In the following, I will thus concentrate on aspects of the paper that they did not discuss in detail, or where I would put another emphasis.

 The present work uses references only scarcely but would benefit from better contextualization, since many aspects of it overlap with work on making DMPs better in general ^[3]^, on integrating them with a broader FAIR ecosystem ^[4]^ and on making them machine actionable ^[5]^.

**Specific comments**

 The study is based on two datasets (cited as references 8 and 11) that have been created and shared in the framework of the project. For the dataset in ref. 11, some methodological details are missing, e.g. the precise source of the DMPs (e.g. a query URL) or the set of instructions (e.g. an API call) used to retrieve and filter the DMPs represented in the collection. Likewise, no version information or URI was given for the H2020 DMP templates (of the European Commission or European Research Council) being discussed.

 Given the "[u]sefulness of the DMP, beyond it being an EC requirement", it would have been instructive to read the DMP for the DMP Use Case Project itself.

 As stated in footnote b, following the four-eyes principle would indeed have been a good choice.

 There are other quality control mechanisms that could have been considered, e.g. the study explicitly focused on beneficiaries of funded proposals, whereas inclusion of DMP-related comments by grant reviewers or by applicants whose proposals were rejected might have added some new insights.

 The filtering of non-ERC DMPs by simple mention of the string "copyright" seems very crude, both because of the overlap with Creative Commons licenses (as alluded to in the discussion), of which some would be compatible with the kind of aggregation and sharing described here, and because some DMPs might express their copyright status using other means (e.g. the copyright sign) or not at all. Because of this, the goal of the filtering - "ensure that only those DMPs which are not IP protected are published" - was likely not achieved with much precision.

 The discussion of these copyright issues (and the uncertainty around the copyright status of ERC DMPs) does highlight the need for stating the copyright and licensing terms in a FAIR manner (for DMPs and other documents, ERC or not), but policy implications like this are only occasionally being touched upon in the present work, even though they would be a useful addition.

 Licensing-related confusion also surfaced in other areas of the text, e.g. in the comment "One project primarily used pre-existing open data. There was therefore no problem in using an open license – however, the business partners in the project were somewhat critical and saw open data more as an obstacle, rather than as an opportunity." Without further context, this is ambiguous at best and needs unpacking - if they "used pre-existing open data", it is hard to understand how they could "[see] open data more as an obstacle, rather than as an opportunity".

 As pointed out by Ron Dekker, some descriptive statistics of the DMP corpus would have been useful. In addition, the presentation of the statistics that are given merits some brushing - is the number of significant digits in the tables really appropriate?

 The need for better contextualization was already pointed out in the general comments, and while this  happens on occasion (e.g. the mention of "OpenAIRE/FAIR Data Expert Group report on the Horizon 2020 template"), here are some more specific examples: the section "Publication of the DMP" could point to the "Exposing Data Management Plans WG" of the Research Data Alliance, whose "Active Data Management Plans IG" would also be relevant for considerations regarding the "Availability of more than one project specific DMP".

 The section "What kind of support is needed and who should provide it?" is focused very much on the DMPs as an end in and of themselves, but misses thoughts regarding how data management plans can actually be leveraged for improved data management, and how the documents containing such plans can be made more readily reusable.

 Hausen and Lanza wrote: "It would be helpful for the reading flow to put the ethical considerations in footnote". I agree that the reading flow is interrupted by having the "Ethical considerations" section between the "Qualitative dimension" and "Quantitative dimension" sections but think the footnotes - at least as implemented in the journal - interrupt the flow even more, and a simple reordering of the sections would suffice.

 Personally, I would like the ethical considerations to be expressed in a more explicit and more FAIR way (e.g. as per https://doi.org/10.5281/zenodo.2559998), but that is clearly beyond the scope of the present paper.

**Linguistic matters**

- There are a number of typos and other issues that need copyediting, e.g. "six cases studies" and "the DMP been dealt with"
- "with was established" (in Ref. 11)
- "creative commons" should be capitalized

If applicable, is the statistical analysis and its interpretation appropriate?

Partly

Is the study design appropriate and is the work technically sound?

Yes

Is the work clearly and accurately presented and does it engage with the current literature?

Partly

Are the conclusions drawn adequately supported by the results?

Yes

Are sufficient details of methods and analysis provided to allow replication by others?

Partly

Are all the source data and materials underlying the results available?

Partly

Reviewer Expertise:

My focus is on the integration between web-based research workflows and research policy, particularly around open science, data management and FAIR data.

I confirm that I have read this submission and believe that I have an appropriate level of expertise to confirm that it is of an acceptable scientific standard, however I have significant reservations, as outlined above.

## References

[1] : Peer Review Report For: Data Management Plans in Horizon 2020: what beneficiaries think and what we can learn from their experience [version 1; peer review: 2 approved]. *Open Research Europe* .2021;1: 10.21956/openreseurope.14412.r26801 10.21956/openreseurope.14412.r26801 PMC1044604137645201

[2] : Peer Review Report For: Data Management Plans in Horizon 2020: what beneficiaries think and what we can learn from their experience [version 1; peer review: 2 approved]. *Open Research Europe* .2021;1: 10.21956/openreseurope.14412.r27179 10.21956/openreseurope.14412.r27179 PMC1044604137645201

[3] : Ten Simple Rules for Creating a Good Data Management Plan. *PLoS Comput Biol* .2015;11(10) : 10.1371/journal.pcbi.1004525 e1004525 10.1371/journal.pcbi.1004525 26492633PMC4619636

[4] Turning FAIR into reality. Final report and action plan from the European Commission expert group on FAIR data.2018; 10.2777/1524 10.2777/1524

[5] : Ten principles for machine-actionable data management plans. *PLoS Comput Biol* .15(3) : 10.1371/journal.pcbi.1006750 e1006750 10.1371/journal.pcbi.1006750 30921316PMC6438441

# Response

**Published:** 2021-10-20

**Authors:** Daniel Spichtinger

**Affiliations:** University of Vienna, Austria

Thank you for your thorough review and for already taking into account the comments from reviewer 1 and 2. I am grateful for the additional suggestions of relevant literature which I will gladly include.

 Generally, the article focuses on this specific project and is not intended as a general discussion of good research data management practice.

 I will get in touch with Uni Wien IT for the technical details requested (query URL, API), since they were in charge of the download. I will add links to the DMP templates.

 As for the inclusion of non-funded proposals and reviewer comments these were not within the scope of the study and would have required significant additional resources (finding out which proposals were rejected); obtaining Evaluation Summary Reports from grant reviewers would also be difficult, since this is not public information. However, these are valuable suggestions for future research.

 Copyright had to be considered, *inter alia to* avoid potential lawsuits of the University of Vienna, in whose repository the list of DMPs was made accessible. However, the reviewer is correct that a mere filtering according to the mention of copyright was indeed a blunt instrument, used also due to the limited resources available. This is why follow up research in a new project (results to be published) has focused on the use of creative commons licences. Future work on copyright related aspect in DMPs is certainly a promising avenue.

 As to the comment on a project using open data but industry partners still seeing open data as an obstacle, this is indeed ambiguous and shows the different approaches and interest even within one and the same project.

 As stated in response to the previous reviewers, statistical information about the DMPs will be added in a separate supplementary file. I will add the policy relevant activities the reviewer mentions.

 As stated in response to a previous reviewer the ethics section is implemented according to the guidelines of ORE.

 Copyediting will be addressed.

# Peer review: Reviewer response for version 1

**DOI:** 10.21956/openreseurope.14412.r27179

**Published:** 2021-07-28

**Authors:** Daniela Adele Hausen, Giacomo Lanza

**Affiliations:** 1 RWTH Aachen University, Aachen, Germany; 2 Physikalisch-Technische Bundesanstalt (PTB), Braunschweig, Germany

The article "Data Management Plans in Horizon 2020: what beneficiaries think and what we can learn from their experience" provides a good preparation and evaluation of the quantitative and qualitative methodology. Its content is very well designed and written in an understandable way. With this contribution, a current topic has been chosen that is well received both in the RDM community and among researchers from all disciplines.

 Nevertheless, we have some suggestions for improving the structure and comprehensibility:

- General:
  - Quantitative information is often reported with nonprecise wording such as “most”, “several”, especially when it comes to the interviews. A quantification of the statements (“5 out of 6”) would be desirable and helpful for the content.
  - Tables are never linked in the text; instead, they should be referred to during presentation of the results.
  - Overall, it should be checked whether a table or even a diagram underlines better the content. From our point of view, it would be very well possible, for example, to present table 2 as a bar chart.
- Page 3, “Introduction”:
  - Openness of research data is a very sensitive topic and is interpreted differently in different disciplines or is not even possible in some cases. Therefore, there are opt-out reasons in the EU and many other funders deal vague with the term openness, so I think openness is formulated too generally.
  - Data FAIRness is a more appropriate request than openness, as it cushions precisely the difficulty of some disciplines just mentioned. Openness can thus emerge in the disciplinary environment and be adapted to the circumstances.
- Page 3, “Qualitative dimension”:
  - A short explanation (2-3 sentences) of the project and the background to the project and the rubric would be very helpful.
  - Concerning the metric from the DART project, please insert the direct link to the presentation / publication and possibly to the relevant page, instead of the repository link.
  - Is there a particular reason to swap the qualitative and quantitative analysis in the methods compared to the order in the results?

- Page 4:
  - It would be helpful for the reading flow to put the ethical considerations in footnote.
  - “CORDIS”: Please provide the link to the website.
  - Results: It would be interesting to get to know where the “prior knowledge of a DMP” came from.
- Pages 4-5:
  - When reading the results, it seems that the terms “data management” and “DMP” are mixed up or are sometimes used in the same way. There is no clear distinction. Please solve this ambiguity.
- Page 5:
  - Table 1: from the fourth row “other” please remove “, namely” or possibly replace it with the additional free-text information collected, if relevant. For rows 1-3 consider shortening the text (e.g. remove “part of”).
  - Figure 1: The legend shows more options than in the diagram. Also, the description of the options can only be guessed in some cases “not available 27” or “others”. Please add also a detailed, self-explaining caption.
- Page 7:
  - DMP project specificities: It would be interesting to get to know which answer is coming from which discipline, in order to fix the first clues of a discipline-specific effect. Also correlations with other side information (project size, partner composition…) might be interesting.
- Page 9:
  - Final thoughts from the interviewees:
    - It would be advisable to discuss these points already here, rather than in the following “discussion and recommendations” section. In particular the very relevant arguments about the "one repository" and “findability and availability of the data”. The findability of DMPs could be improved by assigning to the DMP a persistent identifier such as a DOI or a DataCite DMP ID ( https://support.datacite.org/docs/datacite-dmp-ids).
  - Discussion and recommendations:
    - Some parts are redundant to the above-mentioned results. Our suggestion: Integrate the redundant discussion and further statements above in the respective text passages. In our opinion, the concluding section should convey just the main statements and recommendations in a brief and crispy way.
    - We underline the importance of a one-stop-shop and really like the idea. On the other hand, most universities and research centers already offer inhouse (personal and technical) support to researchers. The best effect could arise with an intelligent coordination of these two offers, exploiting the high acceptance of the inhouse support and the call-specific competence of the EU-Horizon RDM helpdesk service: so one other possibility could be that the EU-Horizon RDM team trains the inhouse support as multiplicators to support researchers in the best way.
- Page 10, “Data availability”:
  - The purpose of the presentation of the used data in the present form is not very clear. Some suggestions to get it a bit clearer:
    - The paragraph “Data availability” normally requires only a concise statement of whether and where the data are available, or which reason speak against data sharing.
    - Additionally, if you wish to describe the data in detail, then please write a concise paragraph for each of the two archives with some relevant information, dropping all redundant information (double file titles, long license statements, …).
    - Additionally, to justify the citation, you are encouraged to recall the underlying data and as references or footnotes in all relevant positions in the previous sections.
    - Since the “interview guide” belongs to the first archive reported, there is no reason to report it separately. That way the distinction between “underlying data” and “extended data” is not applicable and the two sub-titles can be removed.

If applicable, is the statistical analysis and its interpretation appropriate?

Yes

Is the study design appropriate and is the work technically sound?

Yes

Is the work clearly and accurately presented and does it engage with the current literature?

Partly

Are the conclusions drawn adequately supported by the results?

Yes

Are sufficient details of methods and analysis provided to allow replication by others?

Yes

Are all the source data and materials underlying the results available?

Yes

Reviewer Expertise:

DMP, metadata, terminology, data stewardship, research data coordination, active in NFDI4Ing, NFDI4Chem, RDA (DMP groups)

We confirm that we have read this submission and believe that we have an appropriate level of expertise to confirm that it is of an acceptable scientific standard.

# Response

**Published:** 2021-10-20

**Authors:** Daniel Spichtinger

**Affiliations:** University of Vienna, Austria

Thank you for the detailed review and apologies for the late response – due firstly to the fact that I needed to wait for the other reviews and secondly to the fact that the project has finished and it is difficult to find resources to continue the work on the next version of the article.

 “most, several”: The text is meant to further elaborate on the quantitive information provided in the tables, often providing the more qualitative input from the interviews. I will check whether table 2 can be presented differently, since two reviewers have remarked on this.

 EU policy which uses both “openness” and (in recent years) FAIRness and good data management: The article is not intended as an in-depth description of the ECs policies in this regard, but these are detailed in reference 6. A reference to the opt-outs will be added.

 The DART project was eventually not essential to the work undertaken in the study, see also my response to reviewer 1.

 In the methodology, the qualitative dimension is described first since time-wise the project started out with this. The quantitative dimension was then added.

 The ethics paragraph was added on specific request from Open Research Europe. It is therefore not possible to move it to a footnote.

 CORDIS – exact linked need to be checked with Vienna University IT services.

 It is true that some questions referred to data management and some to data management plans. I will review this to make sure that the right terminology for the right question is used.

 Table 1: “namely” will be removed, the other text is important to retain (rows 1-3).

 Figure 1 will be revised, also based on the comments from reviewer 1.

 Discipline specific analysis of the survey responses: this was outside the scope of the project and, given the fact that the project is closed, cannot be implemented at this stage.

 A separate excel file with project information (size, budget) will be provided, as indicated also in the response to reviewer 1.

 Discussions are for the discussions and recommendations section.

 Assigning a persistent identifier to a DMP is a welcome suggestion that will be added to the recommendation’s sections.

 The part on data availability has been written according to the guidance from ORE and will therefore not be changed.

# Peer review: Reviewer response for version 1

**DOI:** 10.21956/openreseurope.14412.r26801

**Published:** 2021-05-11

**Authors:** Ron Dekker

**Affiliations:** 1 Consortium of Social Science Data Archives, Bergen, Norway

The study aims to identify good practices and common challenges when producing Data Management Plans (DMPs).

 The hybrid approach of qualitative and quantitative empirical work is well thought out and pays off in the analysis.

 Some striking results:

- 18% of the DMPs had an own Work Package for data management. However, there is no further analysis whether these projects have 'better DMPs', that is: score higher on the DART items (see below).
- data in one project was not considered as an original work (p. 7). It would have been interesting to elaborate on the (potential) consequences.
- almost 40% of the respondents did not publish their DMP. The author provides some solutions (within EC) but publishing the DMP, assign a DOI to this publication and depositing at Zenodo or similar provides better findability and sustainability.

Still some basic statistics are missing, e.g. size of the projects (budget, partners), size of the DMP (pages, MB), structure of the DMPs. 

 And what ever happened to the scoring of DMPs on the DART rubric (33 items maximum 66 points)? This rubric from the DART project (p.3) could have been explained, incl. the structure of its 33 categories "to standardize the review of data management plans". In addition, the reference could have been directly towards the paper instead of the project website.

 It would have been nice if the EC could have commented on the results, especially the 'Final thoughts'.

 Detailed remarks:

 The name of the Chair of the European Commission is Von der Leyen (p. 3).

 The bullet points on Major challenges (p. 7) are sometimes in capitals and sometimes not. 

 Typo on p. 8 'a lot of shared date' --> '... data'.

 On the selection criteria for the interviews (p. 3): the research interests of the staff ... - although useful - is not a very objective criterion. It would have been nice to see the numbers for each of the filters.

 Figure 1 (p. 5) has five parts, but in the legend there are six categories, incl. an unexplained 'not available 27'.

 In the Use of templates section (p. 6) the reference is to the qualitative survey, but the numbers indicate that this is the quantitative survey.

 At the top of p. 7 it is not explained what the Agency is.

 In the selection there was the criterion of at least two versions of a DMP, but this is not used in the analysis. So why this criterion at all?

 There could have been a check on bias when selecting the DMPs (stage 1 and 2) and on the non-response when going from 840 white listed DMPs to the 108 that responded.

 Table 2 (p. 6) could have been a graphic to facilitate interpretation.

 The second bullet in 'What kind of support is needed' is rather cryptic: what matrix? What should be executed by research support organizations? And the last one: why is the library only relevant for bigger organizations?

 The ARGOS tool is briefly mentioned (p. 9) but not explained, nor whether there are currently other tools to support DMPs, like CESSDA's Data Management Expert Guide.  

If applicable, is the statistical analysis and its interpretation appropriate?

Partly

Is the study design appropriate and is the work technically sound?

Yes

Is the work clearly and accurately presented and does it engage with the current literature?

Yes

Are the conclusions drawn adequately supported by the results?

Yes

Are sufficient details of methods and analysis provided to allow replication by others?

Yes

Are all the source data and materials underlying the results available?

Yes

Reviewer Expertise:

Open science, EOSC, research data infrastructures, data catalogues.

I confirm that I have read this submission and believe that I have an appropriate level of expertise to confirm that it is of an acceptable scientific standard.

# Response

**Published:** 2021-10-20

**Authors:** Daniel Spichtinger

**Affiliations:** University of Vienna, Austria

Thank you for the detailed review and apologies for the very late response – due firstly to the fact that I needed to wait for the other reviews and secondly to the fact that the project has finished and it is difficult to find resources to continue the work on the next version of the article. I agree with your highlighting of salient facts – given that the project as such is closed, these issues would be good follow up projects. As for the statistics that are asked for (size of project, DMP) I will look into providing this as additional information in a separate excel file. I do include the results of the DART analysis as supplementary data. However, this needs to be considered with a “pinch of salt” since, due to the limited resources in the project, only one reviewer (the author) reviewed the selected DMPs. This is why, as the project developed, it was chosen to switch the main emphasis to complementing the qualitative interviews with a quantitative survey. The DART based evaluation, however, was important in that it provided input into the preparation for the qualitative interviews, helping to identify interesting project specifics. In the future it would be preferable to use a “four eyes” approach for such analysis (this is mentioned in footnote b in the article text). The results were also sent to the EC for potential use in their policy making activities.

 Detailed remarks – where I do not provide comments I have implemented them in the main text of the article.

- Figure 1 will be revised.
- The selection criteria of having at least two DMP versions was useful for the qualitative interviews, where this fed into the creation of the interview guide.
  The selection criteria for qualitative interviews was not used in the survey.
- The selection of stage 1 and 2 was based on the objective criteria outlined (is it a DMP? Is it public?) and then undertaken by a number of volunteers.
  The results were also checked by the author. In the view of the author, the existence of systemic bias is therefore unlikely.
- The “cryptic” bullet(s) is/are input from the interviewees. The aim of the paper is not to provide an extensive discussion of the available data management tools.
